# De novo transcriptome sequencing and gene expression profiling of *Magnolia wufengensis* in response to cold stress

**DOI:** 10.1186/s12870-019-1933-5

**Published:** 2019-07-18

**Authors:** Shixin Deng, Jiang Ma, Lili Zhang, Faju Chen, Ziyang Sang, Zhongkui Jia, Luyi Ma

**Affiliations:** 1 Ministry of Education Key Laboratory of Silviculture and Conservation, Forestry College, Beijing Forestry University, Beijing, 100083 People’s Republic of China; 2 School of Landscape Architecture, Beijing Forestry University, Beijing, 100083 People’s Republic of China; 3 Biotechnology Research Center, China Three Gorges University, Yichang, Hubei Province 443002 People’s Republic of China; 4 Forestry Bureau of Wufeng County, Wufeng, Hubei Province 443400 People’s Republic of China

**Keywords:** Cold stress, RNA-Seq, Gene regulation, *Magnolia wufengensis*, Transcriptome

## Abstract

**Background:**

*Magnolia wufengensis* is a new species of *Magnolia* L*.* and has considerable ornamental and economic value due to its unique characteristics. However, because of its characteristic of poor low temperature resistance, *M. wufengensis* is hardly popularization and application in the north of China. Furthermore, the mechanisms of gene regulation and signaling pathways involved in the cold-stress response remained unclear in this species. In order to solve the above-mentioned problems, we performed de novo transcriptome assembly and compared the gene expression under the natural (25 °C) and cold (4 °C) conditions for *M. wufengensis* seedlings.

**Results:**

More than 46 million high-quality clean reads were produced from six samples (RNA was extracted from the leaves) and were used for performing de novo transcriptome assembly. A total of 59,764 non-redundant unigenes with an average length of 899 bp (N50 = 1,110) were generated. Among these unigenes, 31,038 unigenes exhibited significant sequence similarity to known genes, as determined by BLASTx searches (E-value ≤1.0E-05) against the Nr, SwissProt, String, GO, KEGG, and Cluster of COG databases. Based on a comparative transcriptome analysis, 3,910 unigenes were significantly differentially expressed (false discovery rate [FDR] < 0.05 and |log_2_FC (CT/CK)| ≥ 1) in the cold-treated samples, and 2,616 and 1,294 unigenes were up- and down-regulated by cold stress, respectively. Analysis of the expression patterns of 16 differentially expressed genes (DEGs) by quantitative real-time RT-PCR (qRT-PCR) confirmed the accuracy of the RNA-Seq results. Gene Ontology and KEGG pathway functional enrichment analyses allowed us to better understand these differentially expressed unigenes. The most significant transcriptomic changes observed under cold stress were related to plant hormone and signal transduction pathways, primary and secondary metabolism, and photosynthesis. In addition, 113 transcription factors, including members of the AP2-EREBP, bHLH, WRKY, MYB, NAC, HSF, and bZIP families, were identified as cold responsive.

**Conclusion:**

We generated a genome-wide transcript profile of *M. wufengensis* and a de novo-assembled transcriptome that can be used to analyze genes involved in biological processes. In this study, we provide the first report of transcriptome sequencing of cold-stressed *M. wufengensis*. Our findings provide important clues not only for understanding the molecular mechanisms of cold stress in plants but also for introducing cold hardiness into *M. wufengensis.*

**Electronic supplementary material:**

The online version of this article (10.1186/s12870-019-1933-5) contains supplementary material, which is available to authorized users.

## Background

**Fig. 1:**
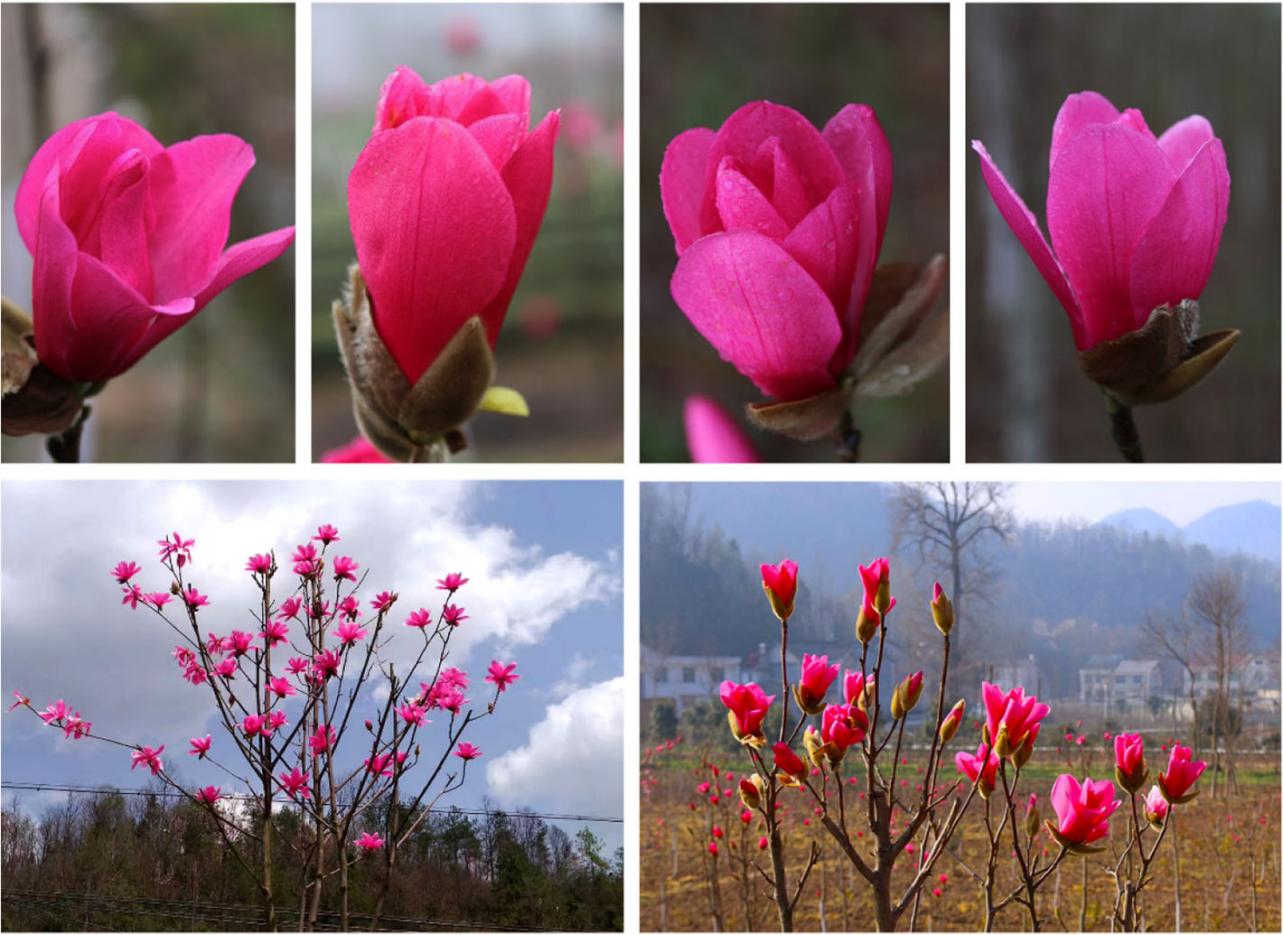
*Magnolia wufengensis* flowers in Wufeng Country, Hubei Province, P. R. China

*Magnolia* is an important landscape tree species around the world. *Magnolia*, which belongs to the subfamily *Magnolioideae* of the family *Magnoliaceae*, is a large genus of approximately 210 flowering plant species. *Magnolia wufengensis* (Fig. 1) is a new species of *Magnolia* exhibiting great ornamental and economic value, with the unique tepal characteristics of evenly red color both inside and outside, flaps of a single color and color uniformity [1]. *M. wufengensis* was first discovered in Wufeng, Hubei Province, southern China [2, 3], and has since been introduced to many cities as an excellent landscaping tree. However, compared with most *Magnolia* species, *M. wufengensis* is more sensitive to chilling injury. This sensitivity to chilling injury leads to unsustainability and low returns regarding *M. wufengensis* seedling production and majorly limits its market value in northern China [4]. To ensure normal growth in cold regions, novel *M. wufengensis* cultivars with improved cold tolerance are needed.

Cold stress is the main form of abiotic stress affecting plant health worldwide and is a major environmental factor limiting plant growth, development, yield and geographical distribution [5]. To overcome this barrier, plants have developed a series of response mechanisms that initiate a battery of events to regulate gene expression, and then physiochemical modifications to enhance their cold resistance [6]. These physiochemical modifications include signaling cascade, modification of the membrane lipid composition, synthesis of cryoprotectant molecules, and an increase in the scavenging activity of reactive oxygen species (ROS) [7–10].

The changes in gene expression triggered by environmental stress are major molecular response mechanisms of plants adapting to environmental challenges [11]. Numerous studies have demonstrated that cold stress leads to global changes in gene expression, and these changes are highly important for transcriptional regulation in plant adaptation to cold stress [12–15]. The changes in the expression of many cold-responsive genes involve both regulatory genes and structural genes [16]. Changes in gene expression lead to the accumulation of many protective proteins, such as heat shock proteins (HSPs) [17], cold-regulated (COR) proteins, dehydrins, cryoprotective proteins [6], and various metabolites [18]. Although numerous cold-responsive genes are continually identified and annotated in arrays of plant species, the number of these genes is still insufficient to elucidate the complete cold-responsive mechanism. Furthermore, genes may be involved in different pathways in different species, and different species may exhibit different cold-responsive genes. Therefore, we must continuously explore cold-responsive regulatory pathways and genes in different species.

Genome-wide transcriptome analysis is an efficient method for detecting and elucidating the breadth of molecular mechanisms involved in physiological processes and thus increasing the efficiency of identifying genes of interest [19]. RNA-Seq technology provides robust methods for genome-wide transcriptome analyses and is increasingly used for obtaining a general overview of RNA transcript profiles in both model and non-model species. Recently, cold-responsive transcriptomes have been studied in many crops and other economically important plants, such as *Prunus dulcis* Mill. [20], *Beta vulgaris* L. [21], *Poncirus trifoliata* (L.) Raf. [19], *Camellia sinensis* [10], and *Eucalyptus dunnii* [22]. However, no studies using such analytical approaches to obtain a comprehensive overview of the genes involved in cold-responsive molecular mechanisms in *M. wufengensis* have been performed thus far.

In order to obtain original insight into the molecular mechanisms underlying the cold response of *M. wufengensis* at the transcriptomic level, we performed transcriptome sequencing and gene expression profiling in normal temperature-treated and cold-treated *M. wufengensis* using the Illumina sequencing technique. As a result, we identified numerous simple sequence repeats (SSRs) and candidate genes that take part in plant development, primary and secondary metabolism, and hormone and signal transduction pathways. This study provides the first report of the *M. wufengensis* response to low-temperature stress. A comprehensive reading of the gene expression changes in leaves under chilling stress can help us understand the cold-stress response pathway at the molecular level and identify efficient ways to enhance cold tolerance in *M. wufengensis.* In addition, the results of this research provide a basis for better understanding the cold responses of many plants because *M. wufengensis* is a basal angiosperm.

## Results

### Response of *M. wufengensis* to chilling stress

**Fig. 2:**
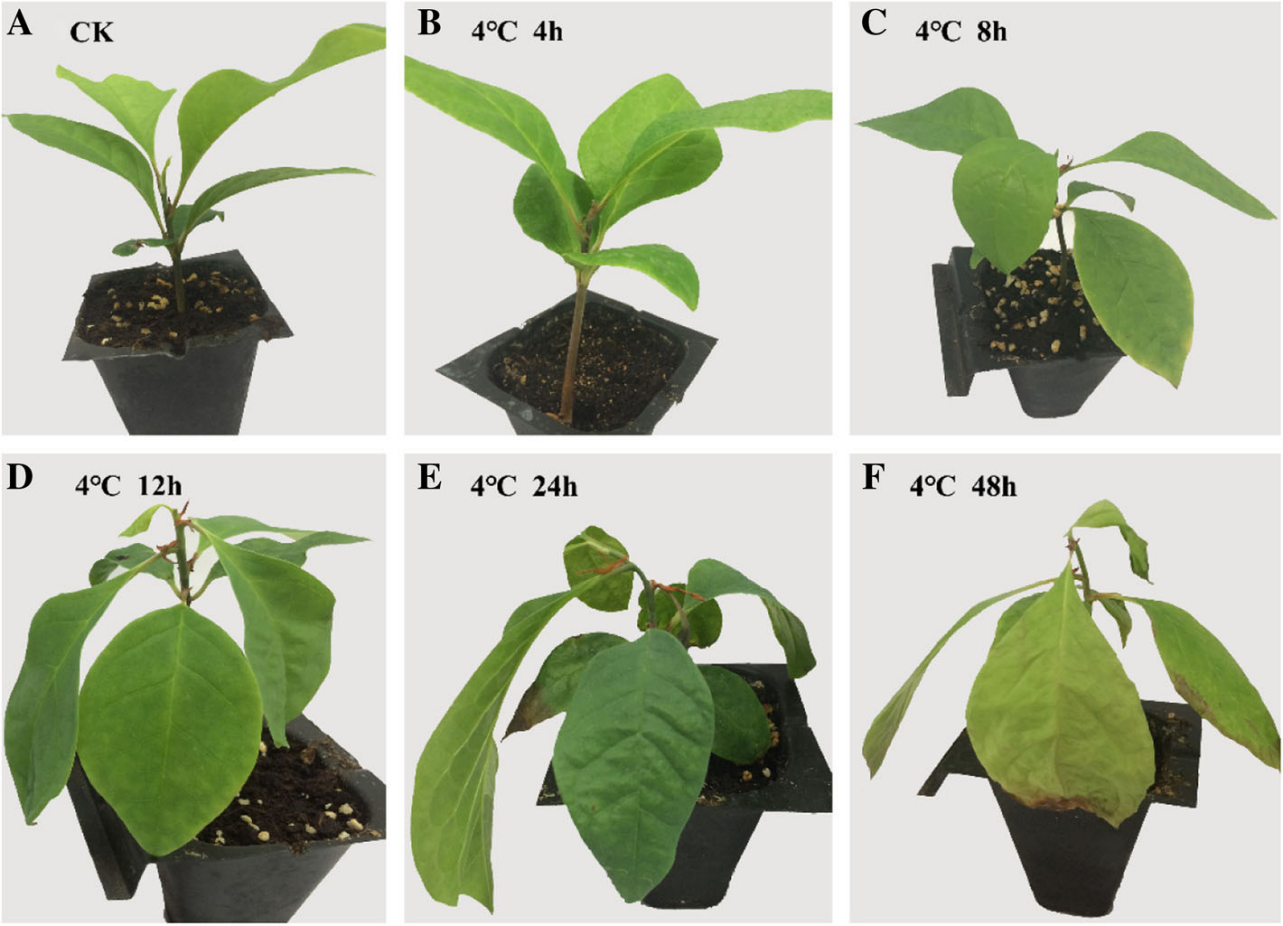
Two-month-old *Magnolia wufengensis* subjected to low-temperature stress (4 °C) in a chamber showing phenotypic changes. **a** Control plant (25 °C). **b** Cold-treated (4 °C) plant at 4 h. **c** Cold-treated (4 °C) plant at 8 h. **d** Cold-treated (4 °C) plant at 12 h. **e** Cold-treated (4 °C) plant at 24 h. **f** Cold-treated (4 °C) plant at 48 h

*M. wufengensis* seeds were collected from Wufeng County in Hubei Province, China and germinated in moistened sand. Under normal conditions, 2-month-old *M. wufengensis* plants exhibit fully expanded mature leaves (Fig. 2a). Following the application of low-temperature treatment at 4 °C, the *M. wufengensis* plants displayed visible morphological changes. No significant changes were initially observed in the plants for 4 h (Fig. 2b). After the plants were subjected to the cold stress for 8 h, the leaves began to show a slight downward trend (Fig. 2c), and after12 h of the low-temperature treatment, the leaves all faced downward and had softened (Fig. 2d). The whole plants began to show evident phenotypic damage as the duration of the cold treatment was prolonged. After 24 h of the low-temperature treatment, the immature stems exhibited loss of strength, and more severely wilted leaves were observed; furthermore, the edges of the leaves began to dry (Fig. 2e). After 48 h of the chilling stress at 4 °C, the leaves had completely withered (Fig. 2f). These results suggest that *M. wufengensis* is likely a cold-sensitive plant.

**Fig. 3:**
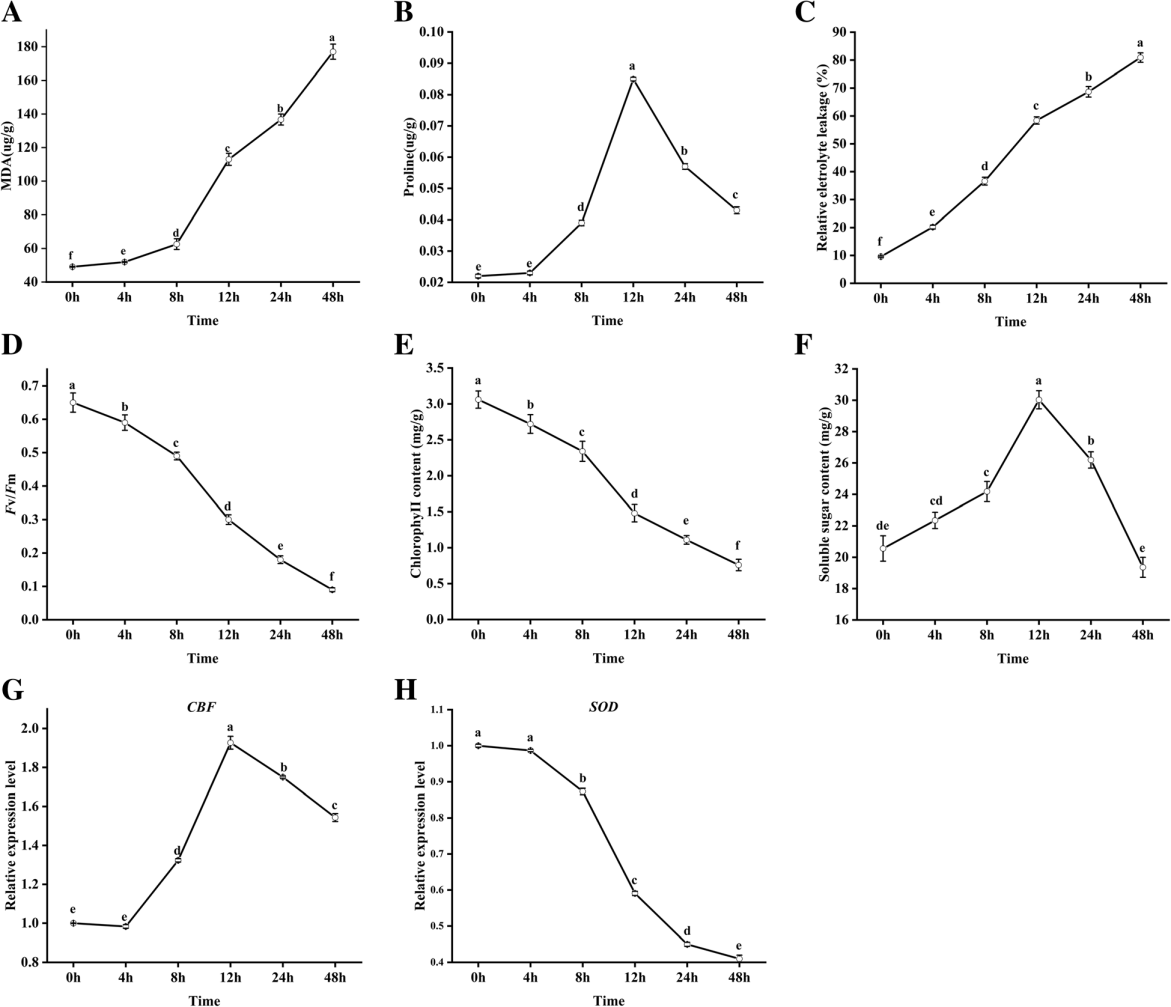
Physiological, biochemical and key gene expression changes in cold-treated *Magnolia wufengensis.* **a** MDA contents. **b** Proline contents. **c** Relative electrolyte leakage changes. **d** The maximum quantum yields of PSII. **e** ChlorophyII content. **f** Soluble sugar content. **g** Expression of *CBF* in *M. wufengensis* leaves exposed to 4 °C for 0 to 48 h. **h** Expression of *SOD* in *M. wufengensis* leaves exposed to 4 °C for 0 to 48 h

In order to determine the ideal time points for transcriptome sequencing, in addition to observing morphological changes, we measured the physiological response of the plants and the changes in the expression of key genes. Malondialdehyde (MDA), which is a final product of lipid peroxidation, is an important physiological index to measure oxidative damage under abiotic and biotic stress [23]; therefore, we tested the MDA content of the leaves during this experiment. In contrast to the control group, the MDA concentration increased as the duration of the cold treatment increased (Fig. 3a) and was highest (showing a nearly 81% increase) after 12 h of the cold-stress treatment, suggesting that the greatest change in gene expression occurred at this time point. The relative eletrolyte leakage also showed similar changes (Fig. 3c). Previous studies have shown that the tolerance of plants to cold and other stresses is closely correlated with the proline and soluble sugar concentration [24]. With extension of the treatment time, the concentration of proline and soluble sugar increased, showing a nearly fivefold and twofold changes after 12 h; although the proline and soluble sugar concentration slightly decreased later, a high concentration was still maintained (Fig. 3b and f). In addition, we also examined the maximum quantum yields of PSII and chlorophyII content and found that these decreased as the duration of the cold treatment increased (Fig. 3d and e), with the maximum decline observed at 12 h. The C-repeat binding factor (CBF)/dehydration responsive element is regarded as one of the most effective pathways in the chilling response of plants [25]. Similar to proline, *CBF* expression reached its highest level at 12 h (Fig. 3g). Superoxide dismutase (SOD) can effectively remove harmful reactive oxygen in plants, which is an important indicator of plant cold resistance [26]. Thus, we examined *SOD* expression and found that it decreased as the duration of the cold treatment increased (Fig. 3h), with the maximum decline observed at 12 h. Based on these results, we chose the samples that had been cold treated for 12 h for transcriptome sequencing.

### Sequencing and de novo transcriptome assembly

Total RNA was extracted from the leaves of 2-month-old *M. wufengensis* from both the control and cold-treated groups (4 °C, 12 h). RNA sequencing of 3 biological replicates from the control and cold-treated samples, designated CK1, CK2, CK3, CT1, CT2 and CT3, was then performed on the Illumina HiSeq 4000 platform. The clean reads were obtained after discarding adaptor sequences, low-quality reads (Q-value < 20) and the reads containing more than 10% ambiguous ‘N’ bases. For the control samples, 86,803,854, 65,817,120, and 61,169,918 clean reads with more than 92% Q30 bases were acquired (Table 1). For the cold-treated samples, 97,721,634, 83,927,960, and 66,717,556 clean reads with more than 93% Q30 bases were acquired (Table 1). The GC content was approximately 48% in the six samples. The biological replicates produced comparable data (Table 1).

**Table 1:** Sequencing the *M. wufengensis* transcriptome in six leaf samples from plants that not cold-treated (CK1, CK2, CK3) or cold-treated (CT1, CT2, CT3)

Sample	Read length	Number of raw reads	Number of clean reads	Size of clean reads (bp)	GC%	Q20 (%)	Q30 (%)
CK1	150 + 150	88,932,026	86,803,854	12,604,279,239	47.50	97.38	92.57
CK2	150 + 150	67,543,450	65,817,120	95,51,610,306	47.84	97.21	92.18
CK3	150 + 150	62,841,084	61,169,918	8,861,064,218	48.19	97.23	92.23
CT1	150 + 150	99,609,824	97,721,634	14,263,070,177	47.90	97.67	93.32
CT2	150 + 150	86,050,574	83,927,960	12,175,957,216	48.55	97.31	92.39
CT3	150 + 150	68,071,038	66,717,556	9,727,205,412	48.32	97.53	92.90

**Fig. 4:**
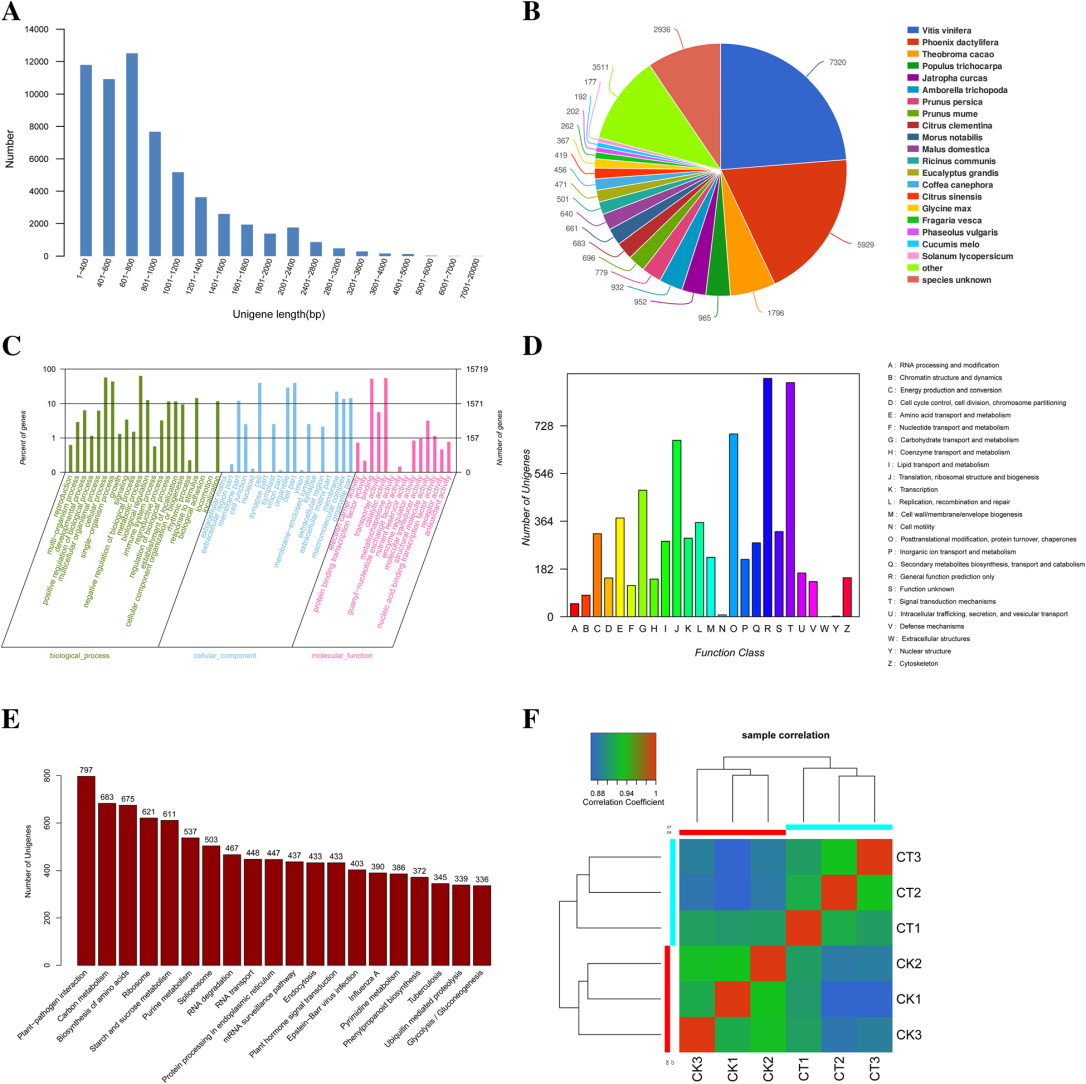
Characteristics of unigenes and samples. **a** Distribution of unigene lengths in *Magnolia wufengensis*. **b** Species distribution of top BLAST hits for each unigene with a cut-off E-value of 1e^− 5^. **c** Functional annotation of unigenes based on Gene Ontology (GO) categorization. **d** Clusters of Orthologous Group (COG) classification. In total, 59,765 unigenes were assigned COG classifications according to the String database and grouped into 25 COG classifications. **e** Top 20 KEGG pathways containing the most unigenes. **f** Correlation analysis between the samples

The filtered clean reads from the six samples were subjected to assembly using the Trinity program [27]. The preliminary assembly generated 94,502 transcripts with an N50 of 1,198 bp. Among these transcripts, the longest transcript was 12,096 bp, and the average transcript length was 916 bp (Table 2). After isoforms were considered, a total of 59,764 unigenes (N50 value =1,110) were generated. The mean size of these unigenes was 899 bp, and their length ranged from 251 bp to 12,096 bp (Table 2). As shown in Fig. 4a, most of the unigenes (55,974, 93.66%) were < 2000 bp in length. In general, the number of unigenes decreased as gene length increased, with lengths between 601 and 800 accounting for the largest proportion (20.41%, 12,198), indicating that the assembly showed high quality.

**Table 2:** Statistics of transcriptome assembly and predicted unigenes

Type	Assembled transcripts	Predicted unigenes
Total sequence number	94,502	59,764
Total sequence base	87,047,615	51,656,716
GC%	42.63	43.16
Largest length (bp)	12,096	12,096
Smallest length (bp)	251	251
Average length (bp)	916	899
N50 length (bp)	1198	1110

### Annotation and classification of *Magnolia wufengensis* unigenes

To identify the putative function of the *M. wufengensis* unigenes, several complementary methods were utilized. The assembled unigenes were searched against the NCBI non-redundant (Nr), SwissProt, String, GO, KEGG, and COG databases using the BLASTX algorithm, with an E-value of less than 1.0 × 10^− 5^. In total, a known protein was found in at least one of the abovementioned databases for 31,038 (51.88%) unigenes (Table 3). Among these unigenes, 30,846 exhibited significant hits (E-value <1e^− 5^) in the Nr database and 20,180 in the GO database. In the other four databases (i.e., SwissProt, KEGG, COG and String), 13,469, 12,180, 7,518 and 13,469 unigenes, respectively, were successfully aligned to known proteins, while 28,756 (48.12%) unigenes did not match any sequences in the six databases. Compared with the other five databases, the Nr database presented the highest proportion of annotations, with 30,846 (99.38%) unigenes being annotated in the Nr database. Overall, the unigene sequences exhibited the most similar BLASTx matches to gene sequences from *Vitis vinifera* (7,320), followed by *Phoenix dactylifera* (5,929), *Theobroma cacao* (1,796), *Populus trichocarpa* (965) and *Jatropha curcas* (952) (Fig. 4b).

**Table 3:** Annotation statistics of *Magnolia wufengensis* unigenes

Database	No. of unigene hits	No. of DEGs
Nr	30,846	2,110
Swisprot	13,469	1,320
KEGG	12,180	700
Gene Ontology	20,180	1,293
COG	7,518	601
String	13,469	988
Total	31,038	2,219

The functions of the *M. wufengensis* unigenes were classified via GO analysis. In total, 15,719 unigenes were successfully categorized into 55 functional groups (Fig. 4c), and these groups were classified into the following three major GO categories using BLAST2GO [28]: ‘biological processes’, ‘cell component’, and ‘molecular function’. The dominant subcategories of the classified genes included ‘metabolic process’ (9,916), ‘cellular process’ (8,873) and ‘single−organism process’ (6,807) in the ‘biological processes’ category; ‘cell’ (6,193), ‘cell part’ (6,193) and ‘organelle’ (4,513) in the ‘cell component’ category; ‘catalytic activity’ (8,519), ‘binding’ (8,171) and ‘transporter activity’ (882) in the ‘molecular function’ category.

The functions of *M. wufengensis* unigenes were predicted and classified by searching the COG database. Assuming that each protein in the COG database independently evolved from an ancestral protein, we classified the 7,342 unigenes based on String annotation into 25 groups of COG classifications (Fig. 4d). Among these classifications, ‘general function prediction only’ (908; 12.37%) accounted for the largest proportion, followed by ‘signal transduction mechanisms’ (892; 12.15%), ‘posttranslational modification, protein turnover, chaperones’ (697; 9.49%) and ‘translation, ribosomal structure and biogenesis’ (673; 9.17%); the remaining unigenes were assigned to the ‘function unknown’ (324; 4.66%) category. However, small clusters were observed in the ‘cell motility’ and ‘nuclear structure’ (7 and 2 unigenes, respectively) classifications. No unigenes were assigned to the ‘extracellular structures’ classification.

To identify the active biological pathways in *M. wufengensis*, pathway annotations of the unigenes was performed using the KEGG pathway tool. The KEGG-annotated unigenes (12,180) were distributed to 341 KEGG pathways (Additional file 1: Table S1); among these pathways, the ‘plant−pathogen interaction’, ‘carbon metabolism’, ‘biosynthesis of amino acids’, ‘ribosome’ and ‘starch and sucrose metabolism’ pathways were the most abundant (Fig. 4e).

### Differentially expressed genes in cold-treated *Magnolia wufengensis* plants

Using the de novo-assembled transcriptome as a reference, the genes expressed in the cold-treated and control groups were identified. We verified consistency among the samples by performing correlation analyses based on FPKM (fragments per kilobase of gene per million mapped reads) values. The high similarity (Fig. 4f, r > 0.91) among the three biological replicates from the control and cold-treated samples demonstrated that the RNA-Seq results were consistent.

To obtain a comprehensive understanding of the transcript expression related to resistance to chilling stress in *M. wufengensis*, we identified the genes that were differentially expressed between the control *M. wufengensis* plants and those grown at cold temperatures. Compared with the control plants, 3,910 significantly differentially expressed genes (DEGs) (false discovery rate [FDR] < 0.05 and |log_2_FC (CT/CK)| ≥ 1) were identified in the cold-treated group using edgeR software. Among these DEGs, the number of up-regulated genes was 2,616, and the number of down-regulated genes was 1,294. Functional annotation of these DEGs was performed using the six databases, and approximately 56.75% (2,218) of the DEGs were successfully annotated (Table 3 and Additional file 2: Table S2).

### GO enrichment analysis of DEGs

To obtain insight into the functional categories of the DEGs induced by cold stress, a GO enrichment analysis was performed using Goatools (Fisher exact test, *P*-value ≤0.05). The most enriched GO category among these DEGs was ‘signal transduction’, followed by ‘hydrolase activity’, ‘lipid metabolic process’, ‘oxidoreductase activity’, ‘carbohydrate metabolic process’, ‘oxidation-reduction process’, ‘response to stimulus’, ‘single-organism metabolic process’, ‘membrane’, and ‘chloroplast’ (Table 4). Thus, the cold resistance process in *M. wufengensis* is complex.

**Table 4:** Significantly enriched GO terms amongst the differentially expressed genes

Gene Ontology	Number of unigenes in whole transcriptome	Number of unigenes differentially expressed	Corrected *P*-value
Signal transduction	304	66	1.33E-07
Hydrolase activity	315	47	7.63E-07
Lipid metabolic process	645	96	1.16E-06
Oxidoreductase activity	1476	154	3.75E-05
Carbohydrate metabolic process	859	87	9.14E-05
Oxidation-reduction process	1271	126	0.000266
Response to stimulus	1705	142	0.000364
Single-organism metabolic process	3876	320	0.00244
Membrane	2053	160	0.0374
Chloroplast	447	34	0.0391

### Pathway enrichment analysis of DEGs

The pathways that displayed significant changes (*P*-value ≤0.05) in response to cold stress were identified using the KEGG database. A total of 16 KEGG pathways were significantly enriched (Table 5), among which the ‘plant hormone signal transduction’, ‘plant-pathogen interaction’, ‘starch and sucrose metabolism’, ‘mitogen-activated protein kinase (MAPK) signaling pathway’, and ‘fatty acid metabolism’ pathways were the most highly represented. The ‘plant hormone signal transduction’ pathway (ko04075) exhibited the most DEGs, suggesting that plant hormones play significant roles in resistance to cold stress in *M. wufengensis*. The second largest number of DEGs were in the ‘plant-pathogen interaction’ category (ko04626), indicating that plants are vulnerable to pathogenic bacteria during exposure to cold stress. The ‘MAPK signaling pathway’ (ko04016) exhibited the fourth largest number of DEGs, indicating that during cold stimulation, the expression of internal genes in plants is regulated by various signaling substances for adaptation to the cold environment. ‘Starch and sucrose metabolism’ (ko00500) and ‘fatty acid metabolism’ (ko01212) were the most highly represented, which was unsurprising because cold causes changes in the metabolism of plants to improve their cold tolerance [29].

**Table 5:** Significantly enriched gene pathways involving differentially expressed genes (DEGs) following the cold stress treatment

Pathway	DEGs with pathway annotation (700)	All genes with pathway annotation (12,180)	*P* value	Pathway ID
CT vs CK				
Plant hormone signal transduction	37 (5.28%)	256 (2.10%)	1.38E-20	ko04075
Plant-pathogen interaction	32 (4.57%)	299 (2.45%)	5.52E-18	ko04626
Starch and sucrose metabolism	28 (4.00%)	280 (2.30%)	5.06E-15	ko00500
MAPK signaling pathway	25 (3.57%)	269 (2.21%)	2.11E-11	ko04016
Fatty acid metabolism	13 (1.86%)	142 (1.17%)	9.70E-11	ko01212
Phenylpropanoid biosynthesis	24 (3.43%)	266 (2.18%)	1.89E-10	ko00940
Cutin, suberine and wax biosynthesis	11(1.57%)	128 (1.05%)	6.46E-10	ko00073
Photosynthesis	13 (1.86%)	177 (1.45%)	4.99E-09	ko00195
Biosynthesis of amino acids	20 (2.86%)	285 (2.34%)	7.83E-09	ko01230
Carbon metabolism	18 (2.57%)	276 (2.27%)	6.22E-08	ko01200
Fatty acid elongation	16 (2.29%)	266 (2.18%)	2.68E-07	ko00062
alpha-Linolenic acid metabolism	18 (2.57%)	315 (2.59%)	4.54E-07	ko00592
Pentose and glucoronate interconversions	13 (1.86%)	236 (1.94%)	5.79E-06	ko00040
Carotenoid biosynthesis	9 (1.29%)	176 (1.44%)	8.09E-06	ko00906
AMPK signaling pathway	11(1.57%)	220 (1.81%)	9.62E-05	ko04152
cAMP signaling pathway	8 (1.14%)	166 (1.36%)	2.61E-04	ko04024

### Validation of RNA-Seq-based DEGs in cold-treated *Magnolia wufengensis* plants by qRT-PCR

**Fig. 5:**
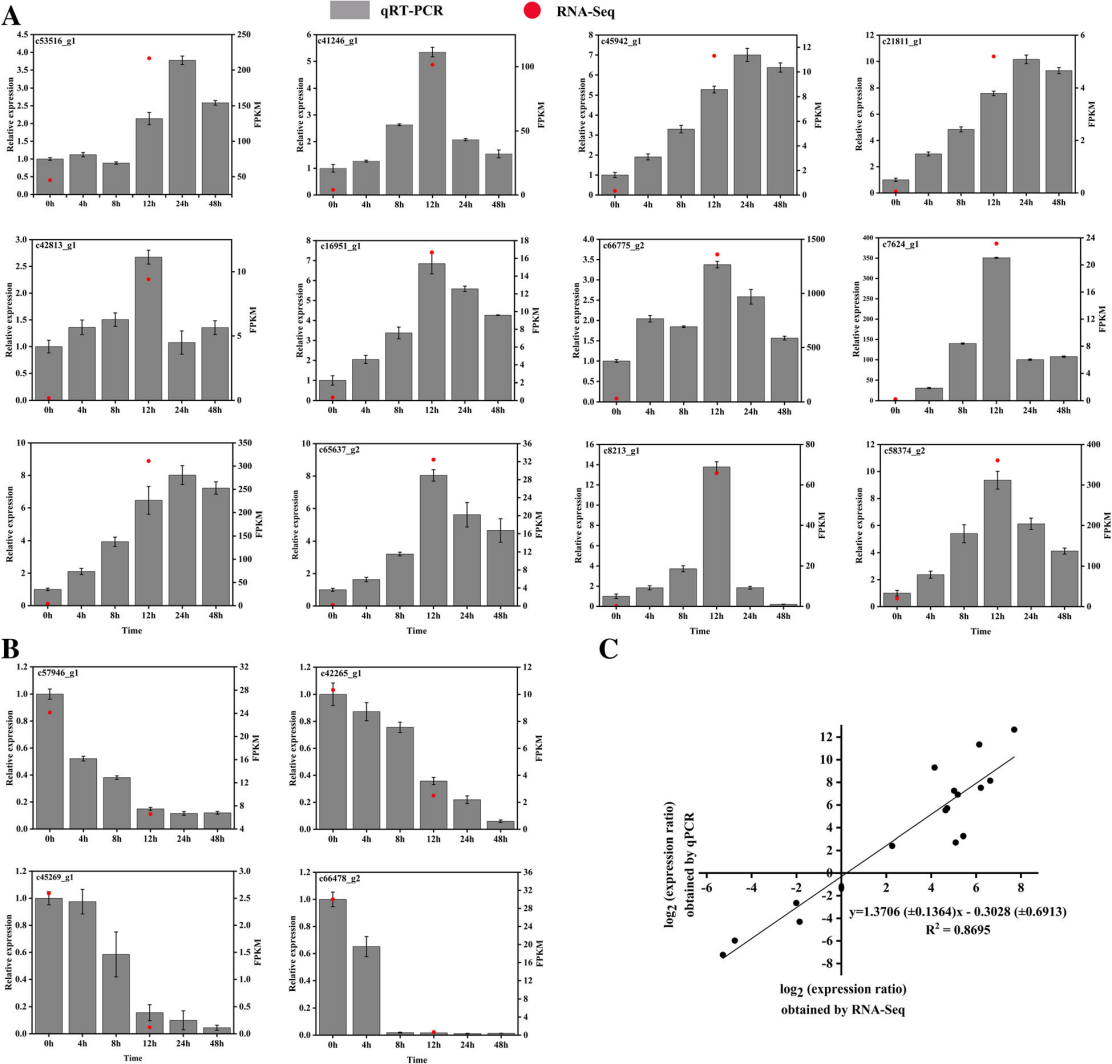
Expression of *M. wufengensis* genes in response to cold at 4 °C for 0 to 48 h as determined by qRT-PCR. **a** Expression of 10 up-regulated genes. **b** Expression of four down-regulated genes. The Y-axis on the left shows the relative gene expression levels analyzed by qPCR (gray histogram), while Y-axis on the right shows corresponding expression data of RNA-Seq (red dot). The X-axis represents the time of 4 °C treatment. **c** Comparison between the log2 of gene expression ratios obtained from RNA-Seq data and qRT-PCR

To verify the accuracy and reproducibility of the RNA-Seq data, we analyzed the transcript abundance of the 16 stochastically selected DEGs using qRT-PCR, including 12 up-regulated genes and 4 down-regulated genes in the unigene dataset (Additional file 2: Table S2). All 12 up-regulated genes were significantly induced by the cold treatment, although their expression changed as the duration of the cold treatment increased (Fig. 5a). Moreover, all four down-regulated genes were correspondingly repressed by the cold treatment, which was in accord with the RNA-Seq results (Fig. 5b). Therefore, the reliability of our RNA-Seq data was confirmed by the consistency between the qRT-PCR results and RNA-Seq analyses.

### Identification of plant hormone and signal transduction-related unigenes

Signal transduction in plants was evidently influenced by cold stress, as verified by both the GO and KEGG enrichment analyses of the DEGs performed in this study. Previous studies have shown that kinases play an essential role in signal transduction by relaying signals via protein phosphorylation [30]. In our dataset, more than 170 genes were predicted to encode protein kinases with varying expression levels in *M. wufengensis* plants under cold-treatment conditions (Additional file 3: Table S3). Genes encoding receptor-like protein kinases (RLKs) accounted for the largest proportion of these genes. More than 67 RLK genes were included in the group of serine/threonine-protein kinases; 21 in the leucine-rich repeat receptor-like protein kinases; eight in the cysteine-rich receptor-like protein kinases; eight in the proline-rich receptor-like protein kinases; and two in the wall-associated receptor kinases (c64483_g1, c66494_g2). Furthermore, the expression of *BRASSINOSTEROID-RESPONSIVE* 1 (BRH1, c41432_g1) decreased, while the expression of an annotated brassinosteroid LRR receptor kinase (c2960_g1) increased under the cold stress. Mitogen-activated protein kinase (MAPK) cascades play important roles in signal transduction induced by abiotic stress [31]. Among the identified DEGs, five MAPKKKs (c64011_g2, c26325_g2, c58822_g1, c20119_g1, c26325_g1) and one MAPKK (c62421_g1) were up-regulated by the cold treatment, while two MAPKKs (c37197_g1, c46922_g1) were down-regulated. In addition, according to the KEGG analysis of the DEGs, the MAPK signaling pathway (ko04016) was induced under the cold treatment conditions, and the expression of several kinases related to the MAPK signaling pathway was also up-regulated.

Calcium, a metal ion, is a secondary messenger in plant cells and plays important roles in many signaling pathways [32, 33]. In plants, oscillations in calcium levels can be monitored by calcium sensor proteins, including calmodulin-like proteins (CML), calmodulin-binding protein (CBL) and calcium-dependent protein kinases (CDPKs), which relay signals and trigger downstream responses [34]. In this study, we identified one gene (c55887_g3) encoding CDPKs, four genes (c66505_g1, c42914_g1, c48548_g1, and c65757_g1) encoding CML, eight genes (c59040_g1, c60069_g1, c62530_g1, c50847_g2, c59040_g2, c50263_g1, c58464_g1, and c46181_g2) encoding CBL and three genes (c53516_g1, c61236_g1, and c61236_g2) encoding CBL-interacting protein kinases (CIPKs) that were up-regulated in the cold-treated group compared with the control group (Additional file 3: Table S3).

According to previous research, reactive oxygen species (ROS) can produce signals in plants triggering cellular changes for resisting changes in the external environment [35]. Plants have an antioxidant system that protects against poisonous oxygen. We identified 36 DEGs encoding enzymes associated with ROS scavenging (Additional file 3: Table S3), including ascorbate peroxidase (APX), peroxidase (POD), glutathione S-transferase (GST), glutathione peroxidase (GPX), polyphenol oxidase (PPO), glutaredoxin, thioredoxin, and polyamine oxidase. Among these antioxidant enzymes, the most important enzymes for ROS scavenging are POD and thioredoxin. ROS promote cellular signaling via an ROS-induced MAPK signaling pathway [31]. In the present study, we identified DEGs that were significantly enriched in the ‘MAPK signaling pathway’ (ko04016, Table 5).

Furthermore, the expression of genes involved in hormone biosynthesis or signal transmission pathways was substantially altered following the cold exposure (Table 6). According to the KEGG analysis of the DEGs, we found that the cold treatment could induce carotenoid biosynthesis (ko00906), which is related to ABA biosynthesis in plants. For instance, compared with the control group, the expression of c52591_g1 was up-regulated 3.93-fold in the cold-treated plants; additionally, the gene predicted to be encoded by c52591_g1 is 9-cis-epoxycarotenoid dioxygenase (NCED), an important enzyme in ABA biosynthesis. Interestingly, the expression of a unigene (c62542_g3) encoding a xanthoxin dehydrogenase, which is an important protein in ABA biosynthesis, was down-regulated by the chilling treatment. Furthermore, two unigenes (c48091_g1and c61792_g2) encoding an ABA 8′-hydroxylase, which is an important enzyme in ABA catabolism, were down-regulated by chilling treatment. This apparent regulation of genes involved in ABA biosynthesis and catabolism suggests the presence of dynamic and multifaceted mechanisms controlling ABA content in response to stress. The changes in the expression of genes involved in the ABA signaling pathway were similar to those related to ABA biosynthesis. For instance, three genes encoding regulatory components of the ABA receptor (RCARs; c60506_g1, c7217_g1, and c38913_g1) were up-regulated, and one RCAR gene (c1769_g1) was down-regulated in response to cold stress. In contrast, one unigene (c62935_g2) encoding PP2C (protein phosphatase 2C), which is a negative regulator of ABA, was down-regulated at 12 h, and three unigenes (c58656_g3, c56328_g1, and c63212_g3) encoding SnRKs (SNF1-related protein kinase) were up-regulated.

**Table 6:** Hormone-related genes that were differentially expressed during cold treatment

Gene ID	Fold change	Annotation	Gene ID	Fold change	Annotation
ABA					
c52591_g1	3.93	9-cis-epoxycarotenoid dioxygenase NCED3	c1769_g1	− 1.53	Regulatory components of ABA receptor 3
c62542_g3	− 1.6	xanthoxin dehydrogenase-like	c62935_g2	−1.53	probable protein phosphatase 2C
c48091_g1	− 3.4	ABA 8′-hydroxylase	c58656_g3	1.45	SNF1-related protein kinase
c60506_g1	1.69	abscisic acid receptor PYL4-like	c56328_g1	2.75	SNF1-related protein kinase regulatory subunit beta-1
c7217_g1	1.05	Abscisic acid receptor PYR1	c63212_g3	1.34	SNF1-related protein kinase catalytic subunit alpha KIN10
c38913_g1	1.77	Regulatory components of ABA receptor 14	c61792_g2	−3.56	ABA 8′-hydroxylase 1
Ethylene					
c50527_g2	−1.52	ethylene receptor 2-like	c53991_g2	−1.88	protein ETHYLENE INSENSITIVE 3-like
c62228_g1	−2.31	EIN3-binding F-box protein 1-like	c47465_g1	−4.65	ethylene-responsive transcription factor 1B-like
c31693_g1	−4	EIN3-binding F-box protein	c57546_g1	2.78	ETHYLENE INSENSITIVE 2 protein
c62228_g4	−2.75	EIN3-binding F-box protein 1-like	c57132_g1	−1.95	ACC oxidase
c53991_g4	−2.29	ETHYLENE INSENSITIVE 3 protein	c53991_g1	−1.93	protein ETHYLENE INSENSITIVE 3-like
JA					
c43502_g1	7.48	4-coumarate--CoA ligase-like 8	c60616_g1	1.89	long chain acyl-CoA synthetase 1
c60697_g4	2.64	Jasmonate O-methyltransferase	c59607_g4	1.35	Jasmonate ZIM domain-containing protein 2
c41372_g1	6.09	4-coumarate--CoA ligase-like 8	c57501_g1	2.74	Jasmonate ZIM domain-containing protein 10
c56341_g2	1.25	Transcription factor MYC	c42250_g1	1.91	transcription factor MYC
Auxin					
c38939_g1	4.68	Auxin transporter-like protein 5	c35279_g1	1.28	auxin response factor 9-like
c3589_g1	1.63	auxin-responsive protein IAA30-like	c57906_g5	1.67	putative auxin response factor 3
c47402_g1	1.42	auxin-induced protein 22D	c46512_g1	1.84	auxin-responsive factor AUX/IAA-related
c48916_g1	4.04	auxin-induced protein IAA6-like	c35732_g2	4.19	Auxin-responsive GH3-like protein 9
c24135_g1	1.43	Auxin-induced protein 22B	c35732_g1	4.04	Auxin-responsive GH3-like protein 11
c48916_g2	2.90	Auxin-induced protein AUX22	c71925_g1	4.17	Auxin-induced protein 15A
c53435_g1	1.48	auxin-responsive protein IAA30-like	c56489_g1	3.11	Auxin responsive protein
c57906_g6	2.51	Auxin response factor 3	c33246_g1	−2.84	auxin-induced protein 6B-like
GA					
c47742_g1	−2.03	Gibberellin receptor GID1C	c1979_g1	2.92	gibberellin-regulated protein 9
c50177_g1	2.37	GRAS family transcription factor			
Salicylic acid					
c60466_g3	−1.35	NPR1–1 protein	c60863_g3	−3.58	pathogenesis-related protein 1
c1824_g2	−3.68	Pathogenesis-related protein PR-1	c59609_g1	−3.15	pathogenesis-related protein PRB1–3-like

Unigenes associated with ethylene-meditated signaling displayed a trend of down-regulation under cold-treatment conditions. Eight ethylene signal transduction-related genes were identified, including one ethylene receptor (ETRs: c50527_g2), three EIN3-binding F-box proteins (EBFs: c62228_g1, c31693_g1, and c62228_g4), three ETHYLENE INSENSITIVE 3 proteins (EIN3: c53991_g4, c53991_g1, and c53991_g2) and one ethylene-responsive transcription factor (ERFs: c47465_g1), and all of these genes were down-regulated in the cold-treated plants. However, one unigene (c57546_g1) encoding the ETHYLENE INSENSITIVE 2 (EIN2) protein was up-regulated.

The expression of the following four genes, which are associated with the biosynthesis of the defense-type hormone jasmonic acid (JA), was up-regulated: one gene encoding long chain acyl-CoA synthetase (c60616_g1), one gene encoding jasmonate O-methyltransferase (c60697_g4) and two genes encoding 4-coumarate--CoA ligase-like (c43502_g1 and c41372_g1). Furthermore, two genes (c59607_g4 and c57501_g1) encoding JAZ and two genes (c56341_g2 and c42250_g1) encoding MYC2, which take part in JA signal transduction, were up-regulated under cold-treatment conditions.

Auxins generally play an important role in plant apical dominance and lateral bud differentiation [36]. Interestingly, we observed significant changes in the 16 genes involved in the auxin signaling pathway, including one gene encoding an auxin transporter-like protein (AUX), six auxin-induced protein IAA6 (AUX/IAA) genes, four auxin response factors (ARF) and two auxin-responsive GH3-like protein (GH3) genes, all of which were up-regulated in the cold-treated plants. In addition, three unigenes encoding SAUR were identified, including two up-regulated unigenes and one down-regulated unigene.

### Identification of transcription factors (TFs) involved in the response to cold stress

Transcription factors (TFs) have important functions in regulating upstream cold signal transduction, which can activate a cascade of downstream gene transcripts [37]. In total, 113 genes encoding TFs belonging to 15 families showed differential expression after 12 h of cold treatment in *M. wufengensis*, including one unclassified gene (c36483_g1) (Additional file 4: Table S4). These TFs, including AP2-EREBP, bHLH (basic helix-loop-helix), MYB (myeloblastosis), MYB-related, NAC (NAM, ATAF1/2, CUC2), bZIP (basic leucine zipper), WRKY (named based on its WRKY amino acid motif), GRAS, and HSF (heat shock transcription factors), are related to resistance to abiotic and biotic stresses and the regulation of plant development. The genes encoding AP2-EREBP and bHLH domain-containing proteins accounted for the largest proportion (17.70%) of these genes, followed by genes encoding WRKY (9.73%), MYB (8.85%), NAC (6.19%), HSF (6.19%), bZIP (5.31%), MYB-related (3.54%) and GRAS (3.54%). *CBFs* were the most represented subfamily, and the expression of two genes (c64671_g1 and c47639_g1) encoding *CBFs* exhibited the greatest changes in our study, presenting increases of 5.51- and 5.22-fold compared with the control plants. Notably, all the bZIP family members were found to be up-regulated in the present study.

### Expression of genes involved in metabolism and biosynthesis

When plants are exposed to cold stress, many of their metabolic processes may exhibit changes. Based on the GO and KEGG pathway enrichment analyses, many DEGs are associated with metabolism and biosynthesis. We found that 28 DEGs (Table 7) were enriched in the ‘starch and sucrose metabolism’ (ko00500) pathway, including 21 up-regulated DEGs and seven down-regulated DEGs. Under cold-treatment conditions, sucrose synthase, pectin methylesterase, glucosidase and pectinesterase inhibitor expression was induced, while the expression of starch synthase and polygalacturonase decreased. Additionally, cold stress increased the abundance of a pectin lyase-like superfamily protein transcript by 6.41-fold.

**Table 7:** Starch and sucrose metabolism-related genes that were differentially expressed during cold treatment

Gene ID	Fold change	Annotation
c48545_g1	3.15	Pectinesterase inhibitor PPE8B
c56058_g1	−1.57	Pectinesterase precursor
c64894_g4	4.88	UDP-glucuronate 4-epimerase 1
c65324_g1	−1.98	Probable galacturonosyltransferase 15
c55207_g1	1.66	Lysosomal beta glucosidase
c65324_g2	−2.05	Glycosyltransferase
c38961_g1	2.53	pectinesterase/pectinesterase inhibitor 39-like
c28537_g1	2.52	sucrose synthase 7-like
c62697_g1	1.38	alpha,alpha-trehalose-phosphate synthase
c43272_g1	2.37	pectinesterase-like
c40566_g1	2.16	Sucrose-UDP glucosyltransferase 7
c65491_g5	−2.08	hypothetical protein
c65060_g5	−1.63	granule-bound starch synthase 2
c64708_g2	1.64	ADP-glucose pyrophosphorylase
c31751_g1	2.77	sucrose synthase 7-like
c64134_g3	1.35	trehalose-6-phosphate synthase
c47120_g1	2.68	pectinesterase/pectinesterase inhibitor PPE8B-like protein
c8515_g1	3.32	Pectin methylesterase 53
c65382_g1	4.01	pectinesterase-like
c65743_g3	−2.25	polygalacturonase-like
c39441_g2	2.28	UDP-glucuronic acid decarbo-xylase 1
c28537_g2	2.58	Sucrose synthase 5
c65060_g3	−1.3	granule-bound starch synthase 2
c14018_g1	−3.8	Polygalacturonase
c53560_g1	1.5	Pectin methylesterase
c65657_g1	2.9	beta-amylase family protein
c49638_g1	6.41	Pectin lyase-like superfamily protein
c64983_g5	−1.41	pectinesterase/pectinesterase inhibitor 12

The ‘lipid metabolism process’ (GO:0006629) was significantly induced by cold stress. A total of 70 genes associated with lipid metabolism were identified, most of which were related to oxylipin, wax, cutin, suberin, sterol, lipid, and fatty acid biosynthetic and metabolic processes (Additional file 5: Table S5). The genes involved in wax biosynthesis were all evidently up-regulated; for example, the expression level of the gene encoding the ADHESION OF CALYX EDGES protein was 8-fold higher in the cold-treated plants than that in the control plants. A notable, approximately 9-fold increase in the gene encoding GDSL esterase/lipase LTL1 (c51965_g1) was observed after cold treatment.

Furthermore, according to the KEGG analysis, secondary metabolic changes occurred after cold treatment, including changes in the ‘phenylpropanoid biosynthesis’ (ko00940) and ‘flavonoid biosynthesis’ pathways (ko00941). In total, 31 DEGs were enriched in the two pathways (Table 8). In the flavonoid biosynthesis pathway, 9 DEGs were identified, and only one gene (c67286_g1) encoding flavonol synthase was down-regulated. In the phenylpropanoid biosynthesis pathway, most up-regulated genes were peroxidases. In addition, dehydrogenase, hydroxylase, methyltransferase and glucosidase were up-regulated. Notably, one caffeoyl-CoA O-methyltransferase (*CCoAMT*; c61212_g1) was up-regulated by 6.21-fold under cold-treatment conditions, and this gene is involved in both pathways.

**Table 8:** Secondary metabolism- related genes that were differentially expressed during cold treatment

Gene ID	Fold change	Annotation
Flavonoid biosynthesis		
c39325_g1	1.2	Caffeoyl-CoA O-methyltransferase
c55700_g1	2.38	Chalcone synthase 1
c61212_g1	6.21	caffeoyl-CoA O-methyltransferase
c62241_g1	2.99	Naringenin-chalcone synthase 3
c62613_g3	2.68	Flavonol 7-O-glucosyltransferase
c27950_g1	2.67	Shikimate hydroxycinnamoyltransferase
c62241_g1	2.99	chalcone synthase
c55433_g2	2.38	Chalcone and stilbene synthases
c67286_g1	−3.55	flavonol synthase
Phenylpropanoid biosynthesis		
c34010_g2	3.5	Cationic peroxidase 1
c4043_g1	4.51	Peroxidase 66 precursor
c40588_g1	2.98	cinnamyl alcohol dehydrogenase 6
c56323_g1	1.81	peroxidase 3
c56553_g1	3.52	Peroxidase 19
c56553_g3	4.5	peroxidase 19
Phenylpropanoid biosynthesis		
c41372_g1	6.09	4-coumarate--CoA ligase
c42717_g1	3.87	peroxidase 46
c51265_g1	1.84	phenylalanine ammonia-lyase
c52904_g1	3.95	peroxidase 72-like
c55207_g1	1.66	Lysosomal beta glucosidase
c61212_g1	6.21	caffeoyl-CoA O-methyltransferase
c66391_g1	2.84	caffeic acid O-methyltransferase
c66566_g1	2.03	ferulate 5-hydroxylase
c55207_g2	1.57	Xylan 1,4-beta-xylosidase
c52526_g1	−4.35	peroxidase 11
c64890_g1	−2.37	cytochrome P450 71A1
c65491_g5	−2.08	hypothetical protein
c65949_g2	−3.5	peroxidase 25
c58388_g1	−3.08	peroxidase N1
c59808_g1	−1.22	peroxidase 15
c60111_g1	−3.59	peroxidase 52

Amino acids are also important for the cold response in plants, as indicated by the ‘biosynthesis of amino acids’ pathway (ko01230). Twenty DEGs were identified in this pathway (Table 9), and most DEGs showed an up-regulated trend. For example, cold treatment increased the expression of glutamate synthase (c62914_g2 and c65821_g6), serine hydroxymethyltransferase (c71333_g1 and c77854_g1) and aspartate kinase (c48954_g1). However, certain genes, such as tryptophan synthase (c40985_g1) and cysteine synthase (c64613_g2), were down-regulated.

**Table 9:** Amino acids biosynthesis-related genes that were differentially expressed during cold treatment

Gene ID	Fold change	Annotation
c31254_g1	2.12	D-isomer specific 2-hydroxyacid dehydrogenase
c34678_g1	−1.94	hypothetical protein
c40985_g1	−4.44	tryptophan synthase beta chain 2
c41899_g1	3.41	phosphoglycerate kinase
c48954_g1	3.96	aspartate kinase family protein
c54342_g1	1.95	nicotianamine aminotransferase A-like isoform X1
c62271_g7	2.49	transaldolase
c62854_g1	−1.69	pyridoxal-5\’-phosphate-dependent enzyme family protein
c62914_g2	1.59	glutamate synthase 1
c63417_g1	−1.38	anthranilate synthase beta subunit 1
c54639_g2	−1.73	branched-chain-amino-acid aminotransferase 3
c58014_g2	3.35	pyridoxal-phosphate dependent enzyme
c59002_g1	2.86	anthranilate phosphoribosyltransferase
c62164_g2	1.51	D-3-phosphoglycerate dehydrogenase
c64613_g2	−1.11	cysteine synthase
c64776_g4	−1.26	glutamine synthetase cytosolic isozyme
c65821_g6	2.02	glutamate synthase
c66224_g3	−1.62	6-phosphofructokinase 6-like
c71333_g1	2.48	serine hydroxymethyltransferase 4
c77854_g1	2.43	serine hydroxymethyltransferase 4

### Cold-responsive genes related to photosynthesis

According to our results, in addition to DEGs related to metabolism, biosynthesis, phytohormones and signal transduction, we also identified many DEGs enriched in the ‘photosynthesis’ (ko00195) and ‘chloroplast’ (GO:0009507) categories. Thus, photosynthesis might be an important process in the cold response of *M. wufengensis*. The chloroplast is a plant cell-specific organelle that supplies most of the plant energy via photosynthesis. Previous studies have shown that cold stress decreases the rate of photosynthesis by destroying the function of many proteins involved in photosynthesis [38]. We identified 46 genes (Additional file 6: Table S6) encoding components of photosystem I (PSI), PSII and chloroplasts that were significantly decreased under cold-treatment conditions in the *M. wufengensis* plants*.* Among these genes, seven genes encoding proteins localized to the thylakoids were identified. For example, *LOX2S* (linoleate 13S-lipoxygenase 2–1), *AOS* (allene oxide synthase), and a hypothetical protein (c48930_g1) were all down-regulated under cold-stress conditions. Additionally, psbQ-like protein 3 (PSb; c42229_g1), chlorophyll A/B binding protein (c66781_g2 and c51541_g1), photosystem I P700 chlorophyll an apoprotein (c61894_g1) and phosphoenolpyruvate carboxylase (c51792_g1) all decreased following cold exposure.

## Discussion

In this study, we described the transcriptional response caused by cold treatment in *M. wufengensis* plants. A comprehensive transcriptomic analysis was performed in both cryogenically treated (4 °C) and untreated plants through Illumina-based 2 × 150 bp paired-end read sequencing. The Illumina a next-generation sequencing platform for transcriptome assembly generates relatively shorter reads but shows a much higher transcriptome coverage at a lower expense than does other platforms [39]. More than 6 Gb of high-quality clean reads were obtained, which were de novo assembled into 59,764 unigenes with an N50 of 1,110 bp, and the average length of the assembled unigenes was 899 bp, indicating that the assembly was of high quality and likely included many full-length cDNAs. Homologous genes were found for 51.88% of the assembled unigenes in at least one of the public databases that was searched, and 51.57% of the sequences presented a homolog that was determined with a high probability score in one of the following four species: *Vitis vinifera* (23.58%)*, Phoenix dactylifera* (19.10%), *Theobroma cacao* (5.78%) and *Populus trichocarpa* (3.11%). Altogether, these results showed that our *M. wufengensis* SRA dataset represents an important transcriptome resource for gene discovery and functional analyses. The cold resistance of plants can be improved by inducing or inhibiting gene expression under cold stress [40, 41]. In this study, 3,910 DEGs were identified, and 45.6% of these DEGs did not exhibit homologous genes in the examined public databases; these genes may either be unique to *M. wufengensis* or represent homologs of genes that have yet to be identified in previous studies in other plants.

### Signaling mediates cold-stress responses

Under stress, plants can trigger the expression of genes involved in multiple signal transduction pathways and further activate downstream regulatory pathways that are associated with physiological adaptation. First, cold stress affects membrane rigidification in plant cells, which is considered to be the primary event that triggers downstream cold stress responses in plants [42]. In our results, we found that 119 genes associated with membrane components were altered (Additional file 8: Table S8), and 91 of them were up-regulated, suggesting enhanced membrane fluidity of *M. wufengensis* under cold stress. The effect of cold on the membrane fluidity and the structural dynamics of membrane proteins initiates cold signaling by affecting ion and metabolite transport [43]. Sixty-one transmembrane transport-related DEGs, including genes associated with organic phosphonate, organic anion, organic acid, amino acid, sulfate and ion transmembrane transport, was identified in cold-treated *M. wufengensis* seedlings (Additional file 9: Table S9). Especially, the release of Ca^2+^ ions from the apoplast, the endomembrane systems, and the organelles has a strong impact on initiating cold acclimation [44]. Intracellular Ca^2+^ sensors, such as CIPKs and calcium-dependent protein kinases (CDPKs), respond to rising cytosolic Ca^2+^-levels [45]. A total of 16 up-regulated Ca^2+^ signaling-related genes were identified under cold-treatment conditions, which is consistent with the expected role of Ca^2+^ in early cold signal transduction. Ca^2+^ induces intracellular biochemical reactions through the sensing and transduction of downstream CDPKs, thereby regulating the plant response to various abiotic stress signals [46]. It is well known CBL proteins interact with a group of serine/threonine protein kinases known as CIPKs [47]. The CBL-CIPK pathway regulates plant responses to various environmental stresses, including cold, drought and salinity stress [48]. CML proteins usually contain four elongation factor (EF)-hand domains to facilitate Ca^2+^ binding [49]. One CDPKs, four CMLs, eight CBLs and three CIPKs were identified as up-regulated in this study, suggesting that the Ca^2+^-mediated signaling pathway has important functions in the *M. wufengensis* response to cold stress. Besides, DEGs contain more than 170 RLKs (Additional file 3: Table S3), which may play an important role in the perception of cold in *M. wufengensis*. Most RLKs contain an extracellular domain, a single transmembrane motif, and a cytoplasmic kinase domain. It is believed that the extracellular domains are involved in cold recognition that subsequently leads to activation of the cytoplasmic kinase domain [50].

ROS are normal products of plant cellular metabolism and frequently accumulate when plants suffer from environmental stress. The excessive production of ROS can cause oxidative damage to plant cells, but ROS are also well-known secondary messengers in various cellular processes in plants, including tolerance to abiotic stresses [35, 51, 52]. The inhibition of photosynthesis is another important consequence of ROS production [53]. In the present study, many DEGs were enriched in the ‘photosynthesis’ (ko00195) and ‘chloroplast’ (GO:0009507) categories. Interestingly, the expression of genes in these pathways decreased after cold treatment, which indicated that the photosynthetic capacity of *M. wufengensis* was inhibited, in turn causing the accumulation of ROS. The delicate balance between ROS production and scavenging determines whether ROS act as damaging or signaling molecules. Excess ROS can be scavenged through an efficient antioxidant system including both nonenzymic and enzymic antioxidants [54]. Genes encoding APX, POD, GST, GPX and PPO were identified in the plants (Additional file 3: Table S3), and previous studies have shown that these genes play important roles in scavenging ROS under abiotic stress [54, 55]. Furthermore, many DEGs were enriched in the carotenoids biosynthesis (ko00906) pathway, studies also have shown that up-regulation of nonenzymic antioxidants, including glutaredoxin, thioredoxin, polyamine and carotenoids, can enhance stress tolerance [56]. The maintenance of a higher antioxidant capacity to scavenge harmful ROS has been shown to be associated with enhanced plant tolerance to environmental stresses [57]. Moderate amount of ROS in *M. wufengensis* mainly play a signaling role, helping to activate the cellular mechanisms associated with stress tolerance [58]. In addition, ROS signals are transduced to downstream targets through the MAPK cascade [59], and a phosphorylation reaction between the upstream receptor and the downstream target is performed by the MAPKs [60]. We observed that the transcript levels of one MAPKK (c62421_g1) and five MAPKKK (c64011_g2, c26325_g2, c58822_g1, c20119_g1, and c26325_g1) were up-regulated by cold treatment. Various components of the ROS signaling transduction pathway may be involved in the regulation of cold-stress responses, and our dataset revealed several potential candidates.

### Phytohormone signals under cold treatment conditions

Phytohormones play key roles and coordinate various signal transduction pathways during the abiotic-stress response [61]. In this study, according to our transcriptome data, the expression of genes involved in signal transduction pathways associated with several plant hormones was altered after cold treatment for 12 h, suggesting that hormone pathways play critical roles in the *M. wufengensis* response to cold stress. ABA is considered a necessary messenger in the plant adaptive response to abiotic stresses. In response to environmental stresses, the level of endogenous ABA rapidly increases, which in turn activates specific signaling pathways and modifies gene expression [62, 63]. The plant response to cold stress has been suggested to be ABA-independent, although contradictory evidence has emerged [64, 65]. NCED, which is a rate-limiting enzyme, is a crucial enzyme in ABA synthesis [66, 67]. Accordingly, we found that an *NCED* gene was up-regulated by 3.93-fold after cold treatment, suggesting that the synthesis of ABA might be elevated in *M. wufengensis* under cold conditions. Furthermore, we detected increased expression of three *RCARs* and *SnRKs* and decreased expression of one *PP2C*, which are key components in the ABA signaling pathway, as follows: *RCARs* are proposed to be ABA receptors; the *PP2C* protein acts as a negative regulator; and *SnRKs* are positive regulators [68, 69]. The presence of ABA leads to the formation of RCAR-PP2C complex, thus inhibiting the activity of *PP2C* [70, 71] and activating SnRKs to act on membrane proteins, ion channels and transcription factors and promote expression of ABA-responsive genes [72]. Thus, ABA-mediated signaling is likely involved in the cold response in *M. wufengensis*.

Ethylene also plays a crucial role in the adaptation and survival of plants under various stress conditions or is the ultimate effector of other hormone-regulated responses [73]. Ethylene signaling has been implicated in the cold-stress response [74, 75], but various studies have shown both positive and negative effects. For instance, many reports have demonstrated the positive role of ethylene in cold stress in rice [76], tomato [74], winter rye [75] and *A. thaliana* [77]. However, Shi et al. [78] reported that ethylene signaling has a negative effect on cold tolerance by inhibiting the expression of *CBF* and type-A *ARR* genes. These contradictory observations may arise from differences in the species studied or the growth of the tissues employed in these studies [77]. In our study, the expression of an ACC oxidase gene (*ACO,* c57132_g1) was found to be repressed following cold exposure. *ACO* is linked to the synthesis of ethylene, and its down-regulation may therefore reduce ethylene production. We also identified 8 genes related to ethylene-meditated signaling that showed a trend of down-regulation. Thus, we hypothesize that ethylene may play a negative role in the cold response of *M. wufengensis.*

The auxins are a group of plant hormones that regulate plant metabolism and are thought to promote plant growth [79]. Interestingly, we found significant changes in the 16 genes involved in the auxin signaling pathway. Although knowledge regarding the role of auxins in abiotic stress is limited, studies are increasingly investigating the role of auxins in cold resistance. One study showed that cold stress primarily targets the transport of intracellular auxin and cannot completely turn off all the auxin protein transport pathways [80]. SIZ1, which is a key regulator of the cold signaling pathway, stabilizes ICE1 under cold conditions by inhibiting its polyubiquitination [81] and has been found to have a negative effect on the phosphate-starvation-induced remodeling of root architecture by controlling auxin patterning [82]. Therefore, whether and how auxin-regulated signaling affects the cold responses in *M. wufengensis* remains to be fully established.

In addition to ABA, ethylene and auxin, other hormones may also affect the plant response to cold stress. The effects of the growth-related hormone GA on abiotic stress and its interaction with other hormones under environmental stress have become increasingly clear [83]. For example, GID1 is a GA receptor that determines GA signaling transduction [84]. The expression of a unigene (c47742_g1) encoding GID1 was observed to be down-regulated in this study, suggesting that GA signaling transduction may be inhibited by cold stress. Thus, cold-stressed plants may conserve energy by slowing their growth in response to adverse conditions. Altogether, different hormonal signals may affect the expression and regulation of many downstream genes.

### Transcription factors involved in the cold-stress response

Transcription factors (TFs) play a critical role in plant development and stress tolerance [40]. These proteins regulate the expression of inducible genes by interacting with cis-acting elements of the promoter region of many stress-inducible genes [85]. Furthermore, TFs highlight the function of diverse transcriptional mechanisms in the stress signaling pathways and are associated with the regulation of various abiotic stress signals and the transcription of various genes [86]. Numerous TF families, including the AP2/EREBP, bHLH, WRKY, MYB, NAC and MYC families, are thought to orchestrate the signals that are transduced when plants are exposed to diverse environmental stresses [87]. According to our results, 113 predicted TFs were induced by cold treatment. The largest group of cold-inducible TFs belonged to the AP2-EREBP, and bHLH family, which was composed of 20 members, respectively. Among these TFs, two unigenes (c64671_g1and c47639_g1) were annotated as *CBF/DREB* genes that have important functions in plant cold tolerance [16]. Therefore, the *CBF* pathway is conserved in the *M. wufengensis* response to cold stress. Many studies have demonstrated that MYB TFs are involved in various cold-stress regulatory pathways, especially in the regulation of *CBF* by the ABA-independent signaling pathway [25]. The NAC family may be involved in the regulation of ABA-dependent signaling pathway genes under diverse stresses, including cold stress [88]. Other TFs, including the bHLH, WRKY, HSF, bZIP, MYB-related, and GRAS families, were also found to be induced by cold stress. Studies have confirmed that members of these families can significantly affect the cold resistance of plants [5], but further study will be required to determine whether these TFs function independently or synergistically to enhance cold resistance in *M. wufengensis*.

### Cold-stress signaling induces cellular protection processes

The complex interactions among diverse signaling pathways and multiple transcription factors collectively affect the physiological responses to cold stress, including the regulation of membranes, cellular redox status, and primary and secondary metabolism [6, 25]. *M. wufengensis* cells rapidly accumulate compatible solutes and osmoprotectants, including sugars, amino acids, fatty acid et al. These osmolytes help to re-establish cellular osmotic balance by increasing the water potential of cells and thereby play a key role in the protection and stabilization of cellular organelles, proteins and membranes [89]. Some studies have confirmed that there is a strong correlation between sugar concentration and cold resistance, and sugar accumulation acts synergistically with cold-inducible gene products [90]. Fructan, sucrose, fructose, and raffinose likely stabilize the membrane system through osmotic regulation and act as ROS scavengers to protect plants from oxidative damage in low-temperature environments [14]. During cold acclimation, sugar level increases under artificial conditions, which could be due to improved transport mediated by sucrose synthase or invertase [91]. These observations are consistent with our results, as greater induction of sugar biosynthesis-related genes was observed in the plants under cold stress. Notably, two genes encoding trehalose synthase (c62697_g1and c64134_g3) were up-regulated, which is consistent with resports by Li et al. [92], who found that plants can counteract abiotic stresses by accumulating trehalose for oxidative detoxification. We also found that the expression of starch synthase and polygalacturonase decreased, likely because most starch breaks down into soluble sugars under cold stress.

Altered lipid biosynthesis, biomembrane rearrangement, and specific fatty acid changes reduce the impairment of cell membranes by cold stress. A group of lipid metabolism-related genes, including genes associated with sterol, cutin, oxylipin, wax, suberin, and fatty acid biosynthesis and metabolism, was identified in cold-treated *M. wufengensis* plants. Wax may play critical roles in regulating the cold resistance of *M. wufengensis*, as evidenced by the high abundance of transcripts of genes related to its biosynthesis. Previous studies have demonstrated that wax biosynthesis and deposition are involved in regulating abiotic stresses tolerance [12]. A gene encoding GDSL esterase/lipase, which contributes to the protection of plants against abiotic stresses [93], was found to be strongly induced under cold stress. This result provides powerful evidence indicating that lipid metabolism in *M. wufengensis* may be crucial in the adaption to cold stress.

A positive correlation has been shown between the accumulation of amino acids and cold tolerance, and amino acids including proline, glutamic acid, glutamine, alanine, aspartic acid, serine and tryptophan have been reported to accumulate in response to low temperatures [94, 95]. Additionally, cold stress can induce osmotic stress, and amino acids act as important osmolytes [96]. In our study, we identified 20 amino acid biosynthesis-related genes that contribute to the protection of *M. wufengensis* plants against cold injury by regulating ion transport and stomatal opening and affecting enzyme activity, gene expression, and redox homeostasis [96]. Thus, the synthesis of amino acids might be promoted to help plants combat cold stress.

In addition to activating the primary mechanisms of sugar accumulation, amino acid biosynthesis and lipid metabolism, secondary mechanisms are induced in plants leading to the biosynthesis of other secondary metabolites, most of which are derived from the phenylpropanoid biosynthesis and flavonoid biosynthesis pathways. Flavonoids prevent oxidative damage under abiotic stress by removing excess ROS [97]. Phenolic compounds, which are the main products of phenylpropanoid biosynthesis, also play various physiological roles in abiotic stress [98]. In this study, 9 flavonoid biosynthesis-related genes were detected, which is similar to the findings reported by Li et al. [41], who demonstrated that the accumulation of flavonoids protects petunias from chilling injury. Among related enzymes in the phenylpropanoid biosynthetic pathway, phenylalanine ammonia-lyase is one of the most relevant [99]. However, we identified only one gene encoding this particular enzyme, whereas most of the identified genes encoded peroxidases, which oxidize phenols and show increased activity in response to various types of stress [100]. Most flavonoids are superior to well-known antioxidants, such as ascorbate, but various flavonoid oxidation processes could also be involved in ROS scavenging [101]. Therefore, peroxidase might significantly modulate these phenomena [102].

### Photosynthesis is inhibited by cold stress

Photosynthesis is the basic physiological process underlying plant growth and development, but low temperatures can significantly affect this process [103]. In our research, we found that photosynthesis was inhibited by cold stress, which inhibited the expression of genes involved in chlorophyll biosynthesis and destroyed the function of the thylakoids. The decline in photosynthesis under cold stress is mainly due to cold stress induced inhibition of the activity of key enzymes associated with photosynthesis [38]. Two photosynthetic enzyme-related genes, encoding linoleate 13S-lipoxygenase 2–1 (*LOX2S*) and allene oxide synthase (*AOS*), were observed to be down-regulated, and PSII was inhibited, as indicated by the low abundance of transcripts of chlorophyll A/B binding protein and psbQ-like protein (*Psb*) genes. In *Elymus nutans*, cold stress can inhibit the expression of photosynthesis-related genes [104]. Furthermore, the inhibition of photosynthesis reduced assimilation on the photosynthetic electron transport chain, which cause redox imblances, support generation of ROS [53], and damage the photosystems [105]. The inhibition of photosynthesis may lead to the accumulation of ROS, resulting in ROS damage, which is likely a factor in the poor cold tolerance of *M. wufengensis*.

## Conclusions

**Fig. 6:**
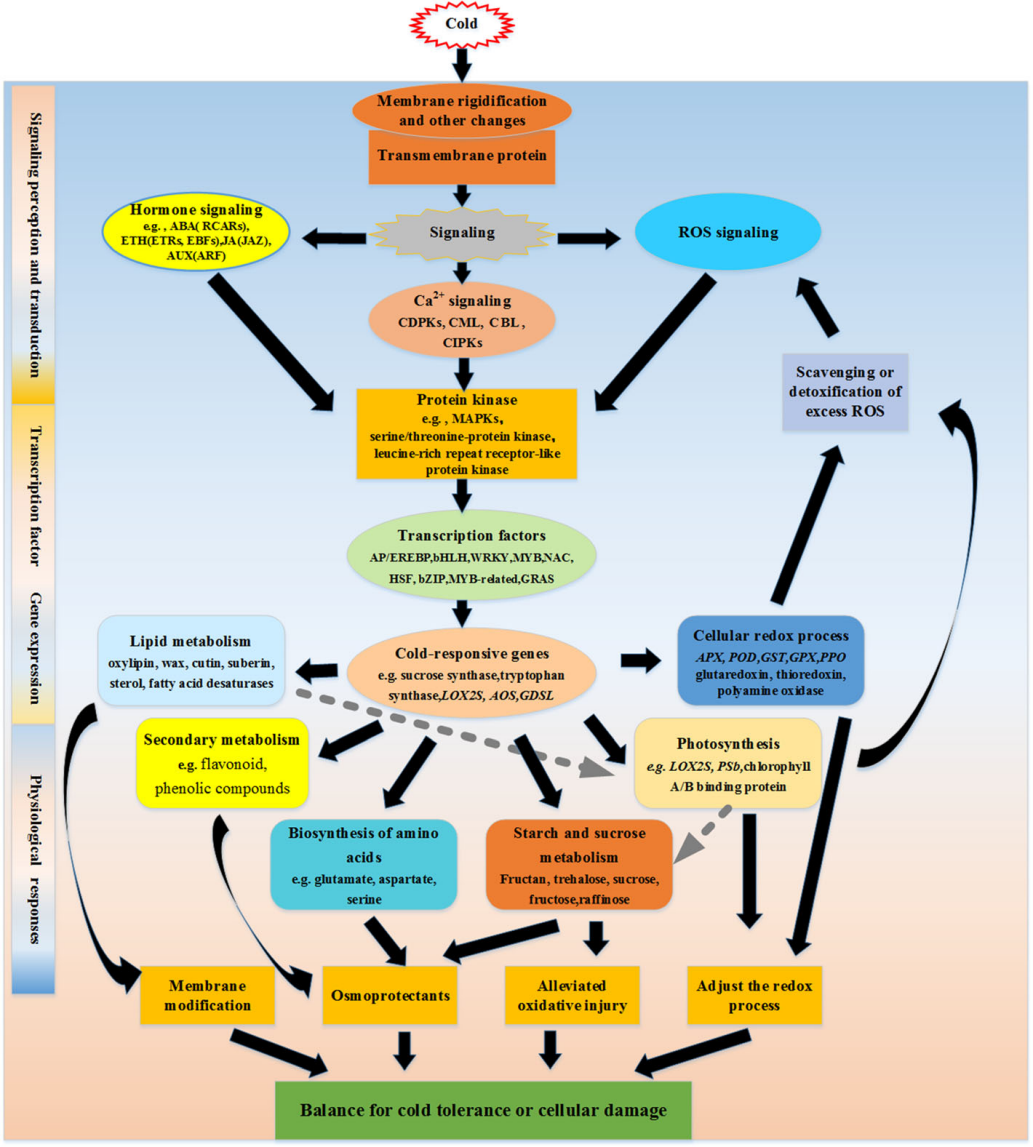
Hypothetical model of the events occurring in the *M. wufengensis* leaves under cold stress

*M. wufengensis* is a new *Magnolia* species with important economic and ornamental value, but it is highly sensitive to low temperatures. In the present study, we comprehensively described the transcriptional responses of *M. wufengensis* leaves to cold stress. Overall, 3,910 DEGs were obtained using RNA-Seq data; these genes included 2,616 up-regulated DEGs and 1,294 down-regulated DEGs. The identified genes were classified by performing functional annotation through BLASTx, GO, and KEGG analyses. Based on the transcriptomic data, a hypothetical model of the response to cold stress in *M. wufengensis* is provided in Fig. 6. Cold stimulation may first be sensed through cold-induced membrane rigidification, most likely via transmembrane proteins, which in turn leads to cold signal transduction, including Ca^2+^, phytohormone and ROS signaling, ultimately inducing changes in cellular metabolism. All signaling pathways act on protein kinases that can switch on various downstream transcription factors (TFs) family proteins. The activation of TFs triggers the expression of a cascade of downstream cold-responsive genes, whose transcription further regulates the homeostasis of cellular metabolism, such as lipid metabolism or secondary metabolism. Furthermore, excess ROS can be scavenged via photosynthesis and cellular redox processes, which further affects ROS signaling. The model established by comparing the differences in gene expression between cold-treated and control plants may promote further studies addressing the molecular mechanisms underlying the cold response in plants. In addition, a large number of the identified genes (approximately 45.65% of all 3,910 DEGs) encode proteins of unknown functions. Studies on these genes might reveal new mechanisms by which *M. wufengensis* responds to cold stress.

## Methods

### Plant materials and stress treatments

The *M. wufengensis* seeds used in this study were collected from Wufeng County in Hubei Province, China. Before the experiment, the seeds should be cleaned and stored at 4 °C in paper bags. First, 0.1% (w/v) sodium hypochlorite were used to sterilize the *M. wufengensis* seeds for 3 min. The seeds were then washed 5 times with sterile distilled water and germinated in moistened sand for 20 d at 25 °C. Next, the seedlings showing uniform growth were transferred to a solid substrate with a mixture of nutritive soil, perlite and vermiculite in a ratio of 5:1:1(v/v), with one seedling per pot. The seedlings were kept in a growth chamber under a 16/8 h (day/night) photoperiod at 25 °C and 65% relative humidity. The cold treatment was performed in a climate chamber under the same photoperiod indicated above at 4 °C. The control group plants were planted under a normal temperature (25 °C), and other conditions were unchanged. When the seedlings were 60 days old, they were exposed to low temperatures. Leaves were collected from the plants after cold treatment for 0 h (control), 4, 8, 12, 24, and 48 h, then immediately frozen in liquid nitrogen and stored at − 80 °C for further analyses. Collection was performed from more than 3 plants at each sampling time, and each sample collection was repeated 3 times to obtain biological replicates.

### RNA extraction

The total RNA was extracted from the leaves of both the cold-treated and control groups using an EASYspin plant RNA Extraction Kit (Aidlab China) following the manufacturer’s instructions. High-quality RNA samples (OD260/280 = 1.8 ~ 2.2, OD260/230 ≥ 2.0, RIN ≥ 6.5, 28S:18S ≥ 1.0, > 10 μg), assessed using a 2100 Bioanalyzer (Agilent) and quantified with an ND-2000 (NanoDrop Technologies), were employed to construct the sequencing library after the removal of genomic DNA using DNase I (TaKara). Each experiment included three biological replicates.

### RNA-Seq library construction and Illumina HiSeq4000 sequencing

The RNA-Seq library was constructed using a TruSeq™ RNA sample preparation kit from Illumina (San Diego, CA). In brief, polyA mRNA was isolated from 5 μg of total RNA using oligo (dT) beads and then fragmented into small pieces with fragmentation buffer. Using these small fragments as templates, double-stranded cDNAs were synthesized with a SuperScript double-stranded cDNA synthesis kit (Invitrogen, CA) and random hexamer primers (Illumina). The double-stranded cDNAs were then end-repaired and phosphorylated, and a polyA tail was added following Illumina’s library construction protocol. Next, target fragments of 200–300 bp were selected via agarose gel electrophoresis and amplified using Phusion DNA polymerase (NEB) over 15 PCR cycles, and the products were used to construct the RNA-Seq library. The final library was quantified by TBS380 fluorometer, and sequencing was carried out on the Illumina HiSeq 4000 platform (2 × 150 bp read length). Each experiment included three biological replicates.

### De novo assembly and sequence annotation

The adaptor reads, low-quality reads (Q-value < 20) and the reads containing more than 10% ambiguous ‘N’ bases were deemed poor-quality reads and discarded, after which the remaining were considered clean reads. Following preprocessing, the clean reads from the control and cold-treated (4 °C for 12 h) 60-day-old plant samples were employed for de novo assembly using Trinity (http://trinityrnaseq.github.io) [106]. The contigs were clustered and further assembled according to paired-end information. The non-terminal extended sequences of contigs identified by Trinity were assembled, and the resulting sequences were defined as unigenes. All the assembled unigenes were searched against public protein databases using BLASTX with an E-value of less than 1.0 × 10^− 5^; the databases included the NCBI nr (NCBI non-redundant protein database), String, SwissProt, COG (Clusters of Orthologous Groups) and KEGG (Kyoto Encyclopedia of Genes and Genomes) databases. The Gene Ontology (GO) annotations of the assembled unigenes were obtained using the BLAST2GO (https://www.blast2go.com/) [28] program with nr annotation. Metabolic pathway analysis of the assembled unigenes was performed according to the KEGG (http://www.genome.jp/kegg/) database [107].

### Analysis and functional enrichment of DEGs

The FPKM (fragments per kilobase of exon per million mapped fragments) method was used to determine the expression level of each transcript. Gene and isoform abundances were calculated with RSEM (http://deweylab.biostat.wisc.edu/rsem/) [108]. Differential expression was analyzed based on the count of the expression level of each transcript in the control and cold-treated samples using EdgeR (Empirical analysis of Digital Gene Expression in R, http://www.bioconductor.org/packages/2.12/bioc/html/edgeR.html) software [109]. A false discovery rate [FDR] < 0.05 and |log_2_FC (CT/CK)| ≥ 1 were used as criteria for identifying significant differences in expression. GO enrichment analyses of differentially expressed genes (DEGs) were performed using Goatools (https://github.com/tanghaibao/Goatools) and KOBAS (http://kobas.cbi.pku.edu.cn/expression.php) [110] with GO annotation. Next, we employed the same method for KEGG pathway functional enrichment analysis of DEGs. The DEGs were considered to be significantly enriched when a Bonferroni-corrected *P*-value ≤0.05 was obtained.

### qRT-PCR analysis of gene expression

Total RNA was extracted from the leaves of 60-day-old plants treated at 4 °C for 0 (control), 4, 8, 12, 24, and 48 h using an EASYspin plant RNA Extraction Kit (Aidlab China). The RNA was then employed to synthesize first-strand cDNAs using an oligo (dT) 18 primer, after the removal of genomic DNA using DNase I (TaKaRa, Japan). qRT-PCR was performed with the StepOnePlus™ Real-Time PCR System (ABI) using SYBR premix Ex Taq (Takara, Japan). Each experiment included three biological replicates. qRT-PCR was performed under the following conditions: 95 °C for 3 min; forty cycles of 95 °C for 15 s, and 58 °C for 30 s. The DNA primers are listed in Additional file 7: Table S7. The relative expression of each gene was calculated according to the comparative cycle threshold (ct) method, and *M. wufengensis ACTION* was employed as a reference.

### Physiological analyses of cold-treated *M. wufengensis* plants

The physiological changes in *M. wufengensis* under the applied cold treatment conditions were analyzed before transcriptome sequencing. The MDA content was determined according to the thiobarbituric acid (TBA) reaction, as proposed by Dhindsa et al. [111] with minor modifications. The proline concentrations were measured using the sulfosalicylic acid-acid ninhydrin method proposed by Bates et al. [112] with slight modifications. Control and cold-treated leaves were also sampled to measure relative eletrolyte leakage [113], soluble sugar concentration [114], maximum quantum yields of PSII [115] and chlorophyII content [116]. Each experiment included three biological replicates.

## Additional files

Additional file 1: **Table S1.** KEGG mapping of the *Magnolia wufengensis* unigenes. (XLSX 206 kb)

Additional file 2: **Table S2.** Differentially expressed genes (DEGs) between the cold treatment and control. FPKM reads per kilobase per million mapped reads. FDR false discovery rate. (XLSX 370 kb)

Additional file 3: **Table S3.** Differentially expressed genes (DEGs) encoding protein kinases and DEGs associated with signaling transduction pathway. (XLSX 26 kb)

Additional file 4: **Table S4.** Differentially expressed transcription factors (TFs) in response to cold treatment of *Magnolia wufengensis* leaves. (XLSX 21 kb)

Additional file 5: **Table S5.** Lipid metabolism-related genes that were differentially expressed during cold treatment. (XLSX 21 kb)

Additional file 6: **Table S6.** Differently-expressed genes involved in photosynthesis in response to cold treatment of *Magnolia wufengensis. (XLSX 13 kb)*

Additional file 7: **Table S7.** Primer information. (XLSX 10 kb)

Additional file 8: **Table S8.** Differentially expressed genes (DEGs) associated with membrane components. (XLSX 14 kb)

Additional file 9: **Table S9.** Transmembrane transport-related genes that were differentially expressed during cold treatment. (XLSX 12 kb)

## Data Availability

The data sets generated or analyzed during this study are included in this published article and its additional files. All the transcriptome data from 6 samples have been deposited in NCBI’s Sequence Read Archive (SRA) under accession number SRP131702 and are accessible through SRA Series (https://trace.ncbi.nlm.nih.gov/Traces/sra/?study=SRP131702).

## Abbreviations

CBF: C-repeat (CRT)-binding factor
COG: Clusters of orthologous groups of protein
DEGs: Differentially expressed genes
FPKM: Fragments per kb per million fragments
GO: Gene ontology
KEGG: Kyoto encyclopedia of genes and genomes
MAPK: Mitogen-activated protein kinase
Nr: Non-redundant protein database
qRT-PCR: Quantitative real-time PCR
ROS: Reactive oxygen species
SwissProt: Annotated protein sequence database
TF: Transcription factor
